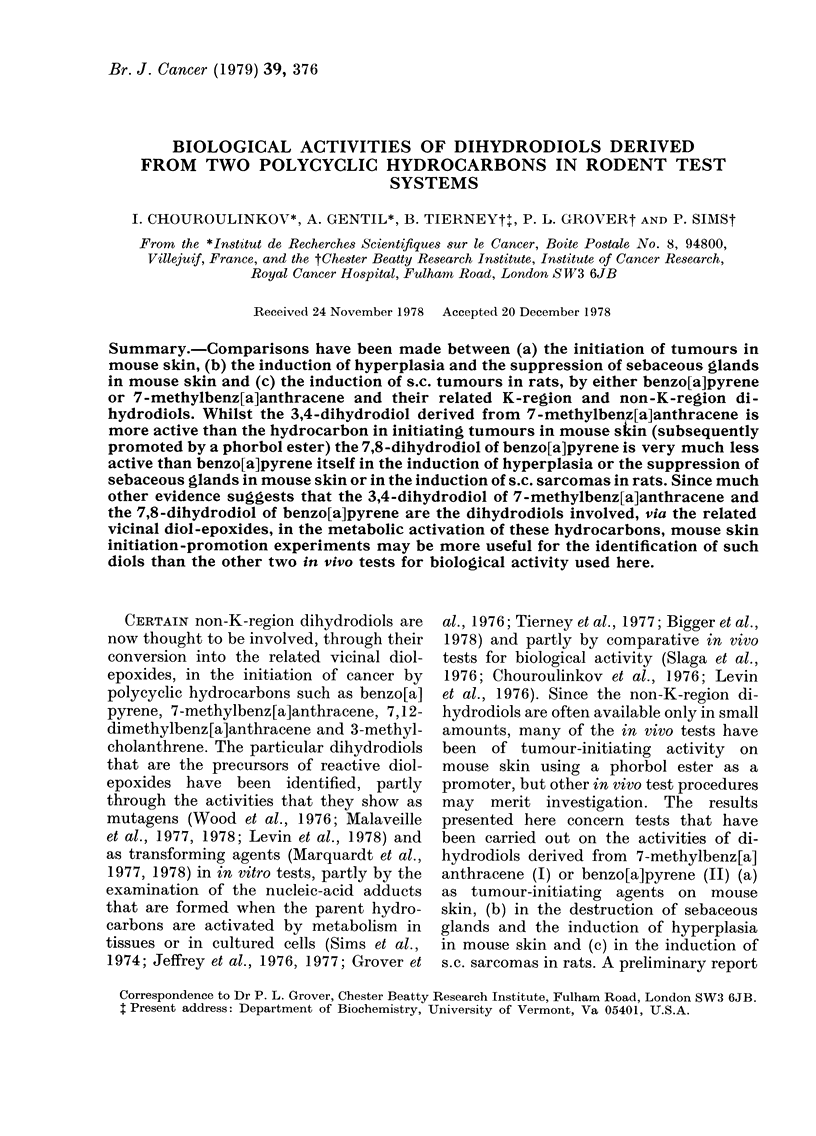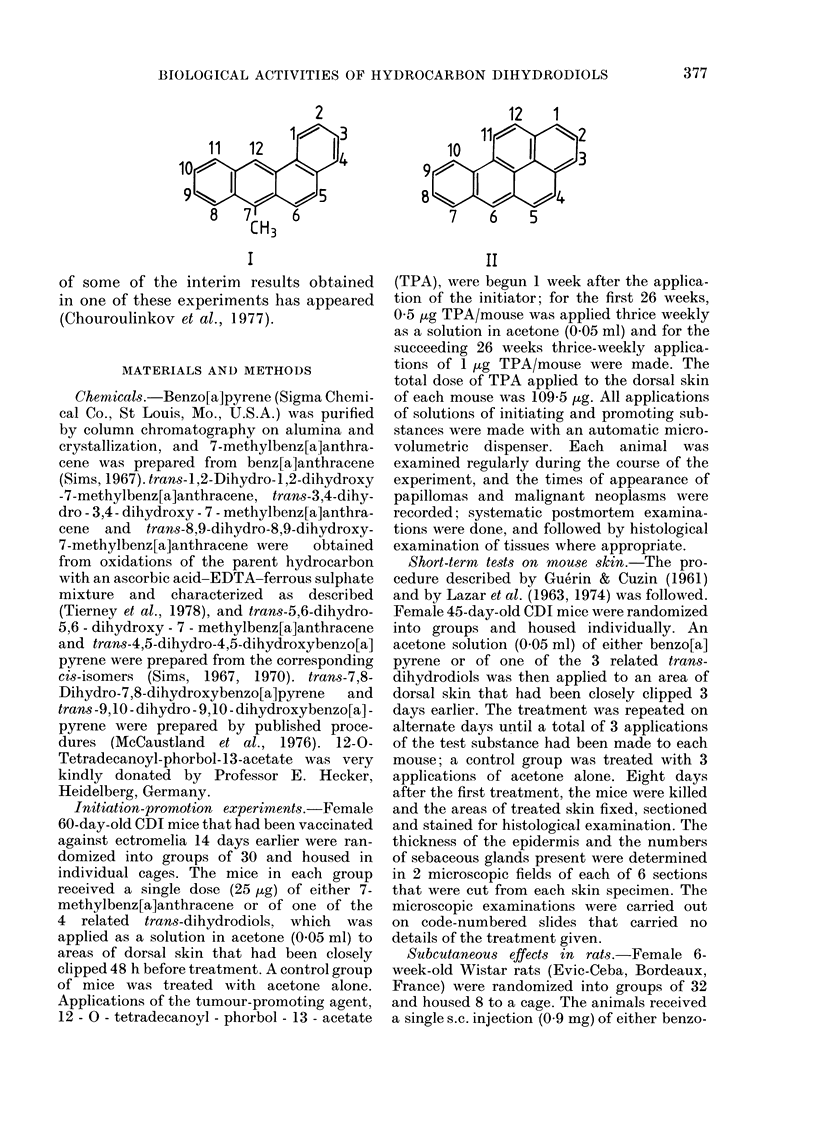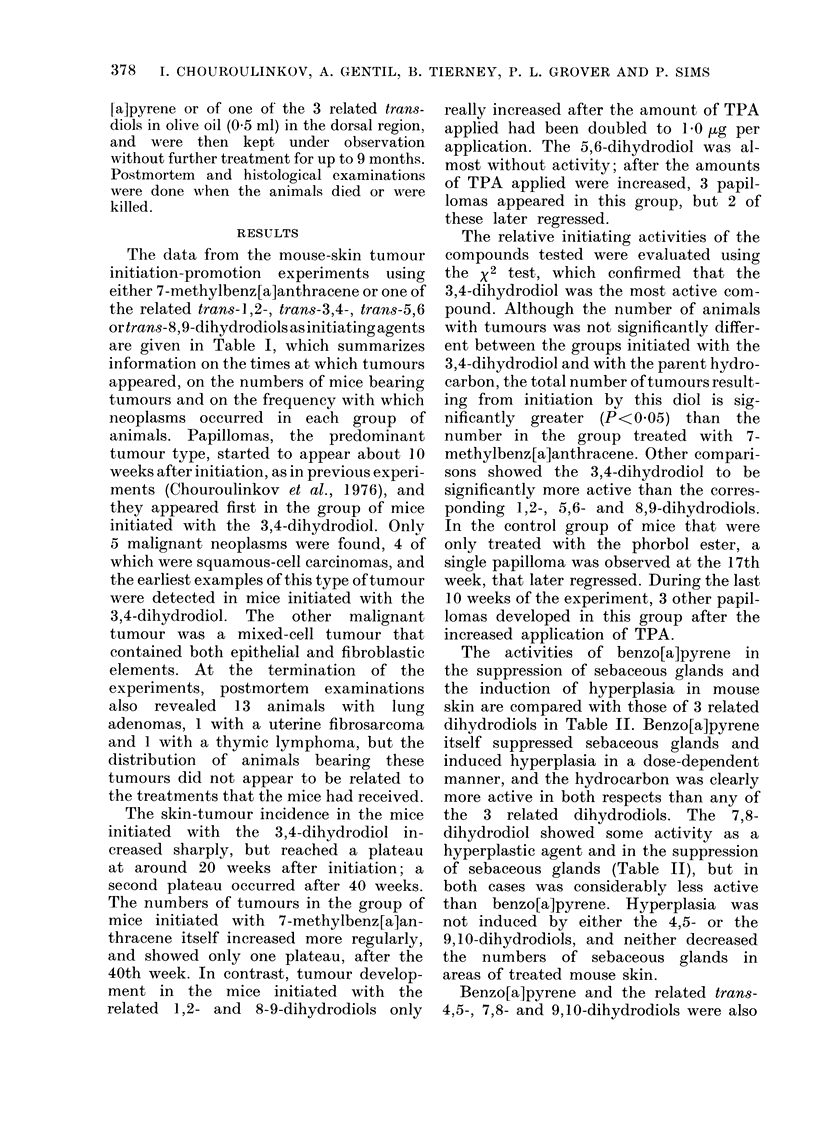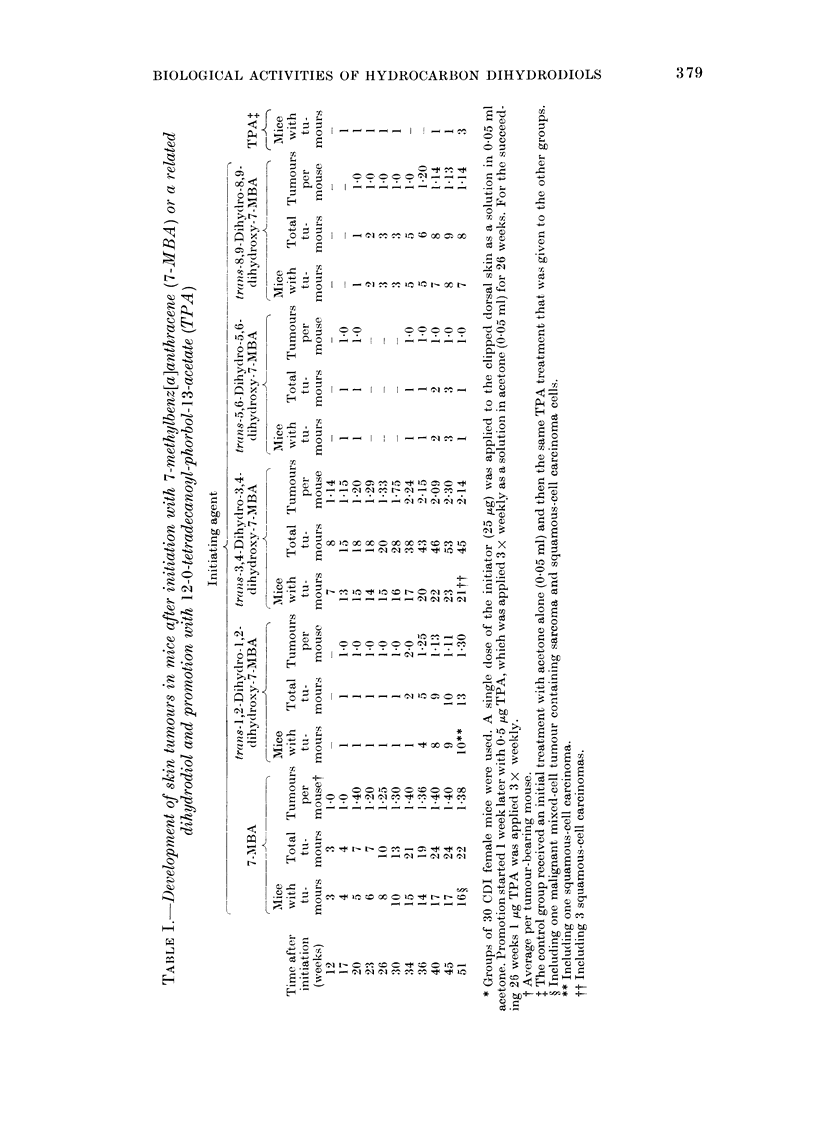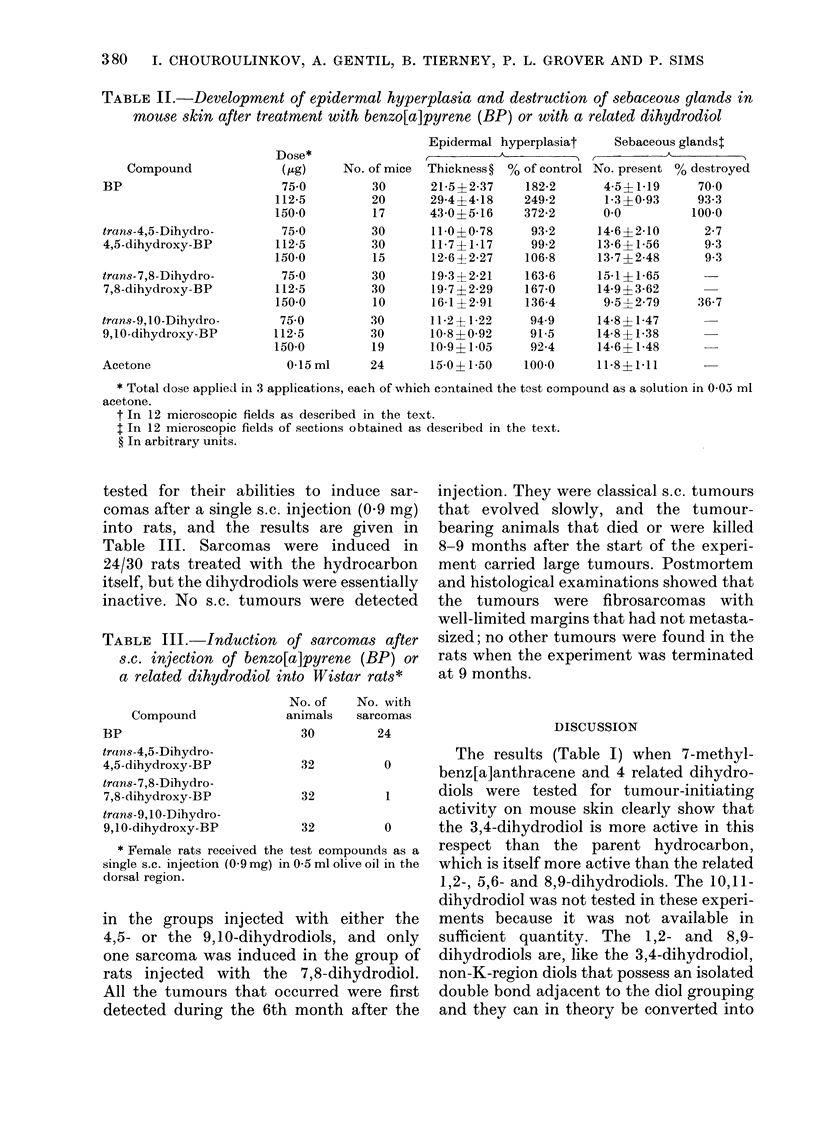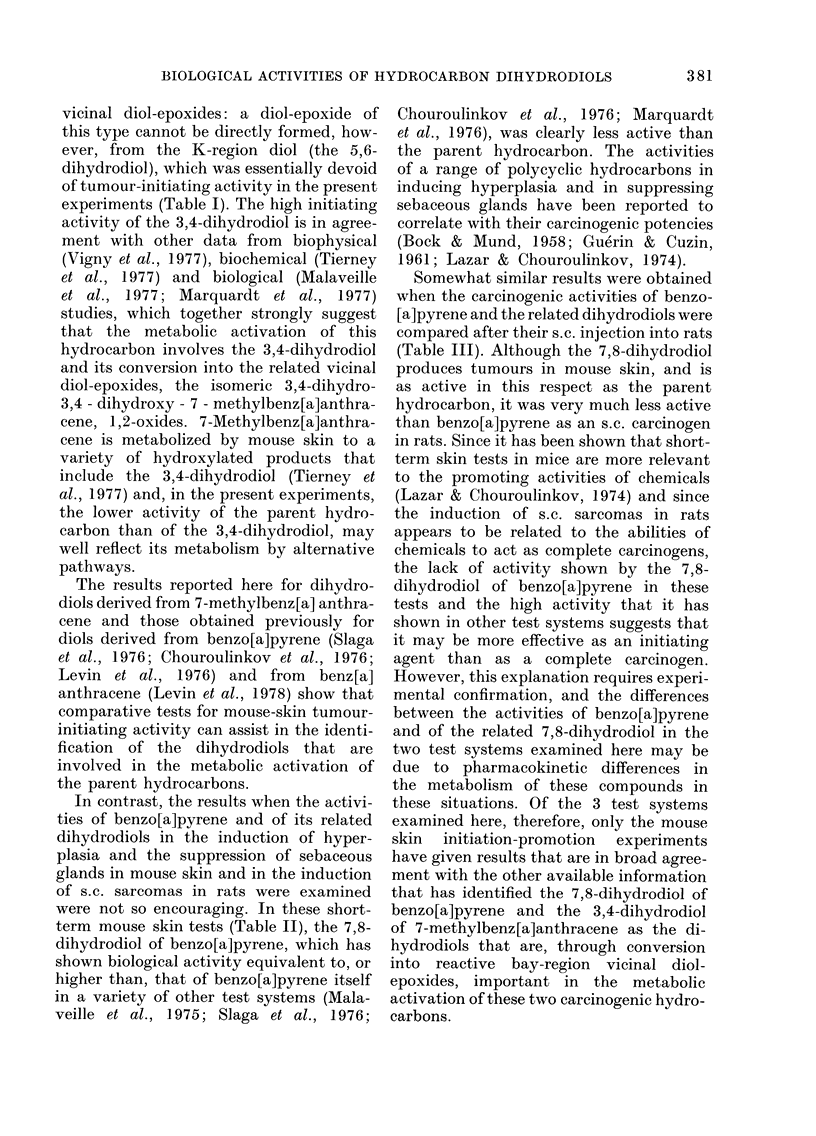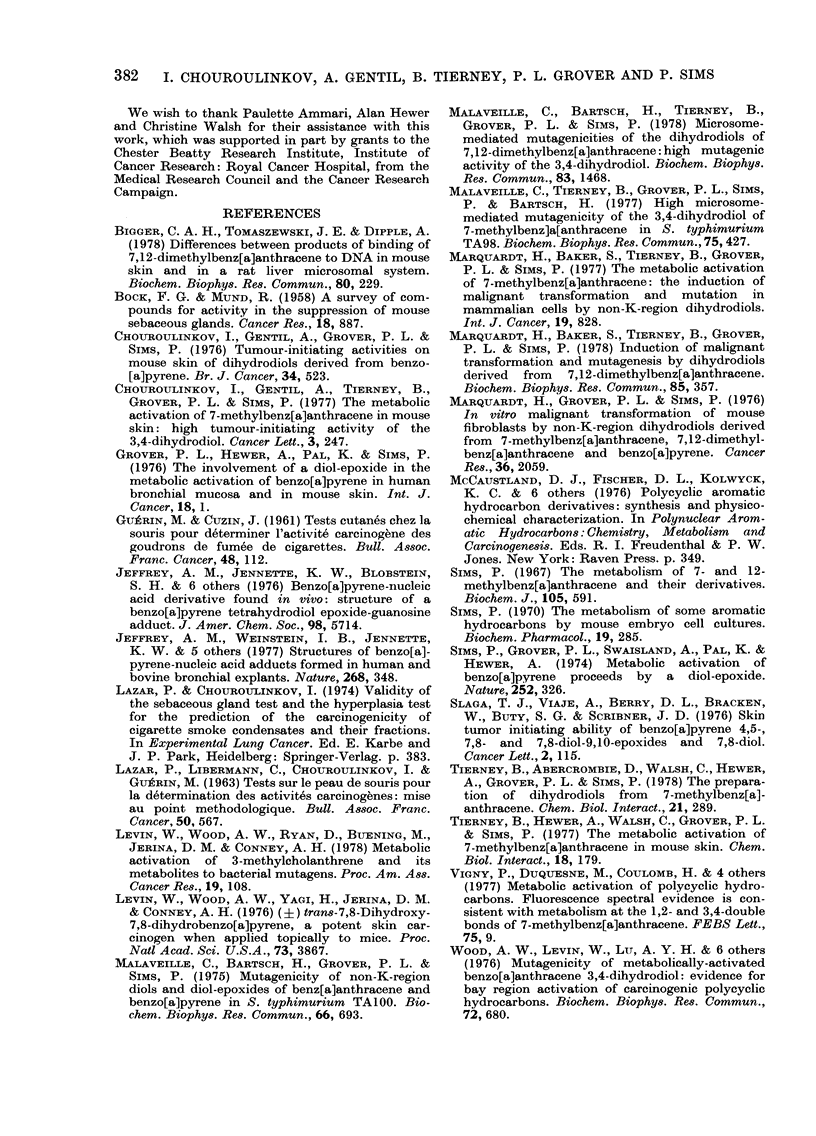# Biological activities of dihydrodiols derived from two polycyclic hydrocarbons in rodent test systems.

**DOI:** 10.1038/bjc.1979.69

**Published:** 1979-04

**Authors:** I. Chouroulinkov, A. Gentil, B. Tierney, P. L. Grover, P. Sims

## Abstract

Comparisons have been made between (a) the initiation of tumours in mouse skin, (b) the induction of hyperplasia and the suppression of sebaceous glands in mouse skin and (c) the induction of s.c. tumours in rats, by either benzo[a]pyrene or 7-methylbenz[a]anthracene and their related K-region and non-K-region dihydrodiols. Whilst the 3,4-dihydrodiol derived from 7-methylbenz[a]anthracene is more active than the hydrocarbon in initiating tumours in mouse skin (subsequently promoted by a phorbol ester) the 7,8-dihydrodiol of benzo[a]pyrene is very much less active than benzo[a]pyrene itself in the induction of hyperplasia or the suppression of sebaceous glands in mouse skin or in the induction of s.c. sarcomas in rats. Since much other evidence suggests that the 3,4-dihydrodiol of 7-methylbenz[a]anthracene and the 7,8-dihydrodiol of benzo[a]pyrene are the dihydrodiols involved, via the related vicinal diol-epoxides, in the metabolic activation of these hydrocarbons, mouse skin initiation-promotion experiments may be more useful for the identification of such diols than the other two in vivo tests for biological activity used here.


*Br. J. Cancer* (1979) **39**, 376

### BIOLOGICAL ACTIVITIES OF DIHYDRODIOLS DERIVED FROM TWO POLYCYCLIC HYDROCARBONS IN RODENT TEST SYSTEMS

I. CHOUROULINKOV*, A. GENTIL*, B. TIERNEY†‡, P. L. GROVER† AND P. SIMS†

*From the *Institut de Recherches Scientifiques sur le Cancer, Boite Postale No.* 8, 94800, *Villejuif, France, and the †Chester Beatty Research Institute, Institute of Cancer Research, Royal Cancer Hospital, Fulham Road, London SW*3 6*JB*

Received 24 November 1978 Accepted 20 December 1978

**Summary.—Comparisons have been made between (a) the initiation of tumours in mouse skin, (b) the induction of hyperplasia and the suppression of sebaceous glands in mouse skin and (c) the induction of s.c. tumours in rats, by either benzo[a]pyrene or 7-methylbenz[a]anthracene and their related K-region and non-K-region dihydrodiols. Whilst the 3,4-dihydrodiol derived from 7-methylbenz[a]anthracene is more active than the hydrocarbon in initiating tumours in mouse skin (subsequently promoted by a phorbol ester) the 7,8-dihydrodiol of benzo[a]pyrene is very much less active than benzo[a]pyrene itself in the induction of hyperplasia or the suppression of sebaceous glands in mouse skin or in the induction of s.c. sarcomas in rats. Since much other evidence suggests that the 3,4-dihydrodiol of 7-methylbenz[a]anthracene and the 7,8-dihydrodiol of benzo[a]pyrene are the dihydrodiols involved, *via* the related vicinal diol-epoxides, in the metabolic activation of these hydrocarbons, mouse skin initiation-promotion experiments may be more useful for the identification of such diols than the other two *in vivo* tests for biological activity used here.**

CERTAIN non-K-region dihydrodiols are now thought to be involved, through their conversion into the related vicinal diol-epoxides, in the initiation of cancer by polycyclic hydrocarbons such as benzo[a]pyrene, 7-methylbenz[a]anthracene, 7,12-dimethylbenz[a]anthracene and 3-methylcholanthrene. The particular dihydrodiols that are the precursors of reactive diol-epoxides have been identified, partly through the activities that they show as mutagens (Wood *et al.*, 1976; Malaveille *et al.*, 1977, 1978; Levin *et al.*, 1978) and as transforming agents (Marquardt *et al.*, 1977, 1978) in *in vitro* tests, partly by the examination of the nucleic-acid adducts that are formed when the parent hydrocarbons are activated by metabolism in tissues or in cultured cells (Sims *et al.*, 1974; Jeffrey *et al.*, 1976, 1977; Grover *et al.*, 1976; Tierney *et al.*, 1977; Bigger *et al.*, 1978) and partly by comparative *in vivo* tests for biological activity (Slaga *et al.*, 1976; Chouroulinkov *et al.*, 1976; Levin *et al.*, 1976). Since the non-K-region dihydrodiols are often available only in small amounts, many of the *in vivo* tests have been of tumour-initiating activity on mouse skin using a phorbol ester as a promoter, but other *in vivo* test procedures may merit investigation. The results presented here concern tests that have been carried out on the activities of dihydrodiols derived from 7-methylbenz[a]anthracene (I) or benzo[a]pyrene (II) (a) as tumour-initiating agents on mouse skin, (b) in the destruction of sebaceous glands and the induction of hyperplasia in mouse skin and (c) in the induction of s.c. sarcomas in rats. A preliminary report

Correspondence to Dr P. L. Grover, Chester Beatty Research Institute, Fulham Road, London SW3 6JB.
‡ Present address: Department of Biochemistry, University of Vermont, Va 05401, U.S.A.

I

II

of some of the interim results obtained in one of these experiments has appeared (Chouroulinkov *et al.*, 1977).

MATERIALS AND METHODS

*Chemicals.*—Benzo[a]pyrene (Sigma Chemical Co., St Louis, Mo., U.S.A.) was purified by column chromatography on alumina and crystallization, and 7-methylbenz[a]anthracene was prepared from benz[a]anthracene (Sims, 1967). *trans*-1,2-Dihydro-1,2-dihydroxy-7-methylbenz[a]anthracene, *trans*-3,4-dihydro-3,4-dihydroxy-7-methylbenz[a]anthracene and *trans*-8,9-dihydro-8,9-dihydroxy-7-methylbenz[a]anthracene were obtained from oxidations of the parent hydrocarbon with an ascorbic acid–EDTA–ferrous sulphate mixture and characterized as described (Tierney *et al.*, 1978), and *trans*-5,6-dihydro-5,6-dihydroxy-7-methylbenz[a]anthracene and *trans*-4,5-dihydro-4,5-dihydroxybenzo[a]pyrene were prepared from the corresponding *cis*-isomers (Sims, 1967, 1970). *trans*-7,8-Dihydro-7,8-dihydroxybenzo[a]pyrene and *trans*-9,10-dihydro-9,10-dihydroxybenzo[a]-pyrene were prepared by published procedures (McCaustland *et al.*, 1976). 12-O-Tetradecanoyl-phorbol-13-acetate was very kindly donated by Professor E. Hecker, Heidelberg, Germany.

*Initiation-promotion experiments.*—Female 60-day-old CDI mice that had been vaccinated against ectromelia 14 days earlier were randomized into groups of 30 and housed in individual cages. The mice in each group received a single dose (25 μg) of either 7-methylbenz[a]anthracene or of one of the 4 related *trans*-dihydrodiols, which was applied as a solution in acetone (0·05 ml) to areas of dorsal skin that had been closely clipped 48 h before treatment. A control group of mice was treated with acetone alone. Applications of the tumour-promoting agent, 12-O-tetradecanoyl-phorbol-13-acetate (TPA), were begun 1 week after the application of the initiator; for the first 26 weeks, 0·5 μg TPA/mouse was applied thrice weekly as a solution in acetone (0·05 ml) and for the succeeding 26 weeks thrice-weekly applications of 1 μg TPA/mouse were made. The total dose of TPA applied to the dorsal skin of each mouse was 109·5 μg. All applications of solutions of initiating and promoting substances were made with an automatic micro-volumetric dispenser. Each animal was examined regularly during the course of the experiment, and the times of appearance of papillomas and malignant neoplasms were recorded; systematic postmortem examinations were done, and followed by histological examination of tissues where appropriate.

*Short-term tests on mouse skin.*—The procedure described by Guèrin & Cuzin (1961) and by Lazar *et al.* (1963, 1974) was followed. Female 45-day-old CDI mice were randomized into groups and housed individually. An acetone solution (0·05 ml) of either benzo[a] pyrene or of one of the 3 related *trans*-dihydrodiols was then applied to an area of dorsal skin that had been closely clipped 3 days earlier. The treatment was repeated on alternate days until a total of 3 applications of the test substance had been made to each mouse; a control group was treated with 3 applications of acetone alone. Eight days after the first treatment, the mice were killed and the areas of treated skin fixed, sectioned and stained for histological examination. The thickness of the epidermis and the numbers of sebaceous glands present were determined in 2 microscopic fields of each of 6 sections that were cut from each skin specimen. The microscopic examinations were carried out on code-numbered slides that carried no details of the treatment given.

*Subcutaneous effects in rats.*—Female 6-week-old Wistar rats (Evic-Ceba, Bordeaux, France) were randomized into groups of 32 and housed 8 to a cage. The animals received a single s.c. injection (0·9 mg) of either benzo-

[a]pyrene or of one of the 3 related *trans*-diols in olive oil (0·5 ml) in the dorsal region, and were then kept under observation without further treatment for up to 9 months. Postmortem and histological examinations were done when the animals died or were killed.

### RESULTS

The data from the mouse-skin tumour initiation-promotion experiments using either 7-methylbenz[a]anthracene or one of the related *trans*-1,2-, *trans*-3,4-, *trans*-5,6 or *trans*-8,9-dihydrodiols as initiating agents are given in Table I, which summarizes information on the times at which tumours appeared, on the numbers of mice bearing tumours and on the frequency with which neoplasms occurred in each group of animals. Papillomas, the predominant tumour type, started to appear about 10 weeks after initiation, as in previous experiments (Chouroulinkov *et al.*, 1976), and they appeared first in the group of mice initiated with the 3,4-dihydrodiol. Only 5 malignant neoplasms were found, 4 of which were squamous-cell carcinomas, and the earliest examples of this type of tumour were detected in mice initiated with the 3,4-dihydrodiol. The other malignant tumour was a mixed-cell tumour that contained both epithelial and fibroblastic elements. At the termination of the experiments, postmortem examinations also revealed 13 animals with lung adenomas, 1 with a uterine fibrosarcoma and 1 with a thymic lymphoma, but the distribution of animals bearing these tumours did not appear to be related to the treatments that the mice had received.

The skin-tumour incidence in the mice initiated with the 3,4-dihydrodiol increased sharply, but reached a plateau at around 20 weeks after initiation; a second plateau occurred after 40 weeks. The numbers of tumours in the group of mice initiated with 7-methylbenz[a]anthracene itself increased more regularly, and showed only one plateau, after the 40th week. In contrast, tumour development in the mice initiated with the related 1,2- and 8-9-dihydrodiols only really increased after the amount of TPA applied had been doubled to 1·0 $\mu$g per application. The 5,6-dihydrodiol was almost without activity; after the amounts of TPA applied were increased, 3 papillomas appeared in this group, but 2 of these later regressed.

The relative initiating activities of the compounds tested were evaluated using the $\chi^2$ test, which confirmed that the 3,4-dihydrodiol was the most active compound. Although the number of animals with tumours was not significantly different between the groups initiated with the 3,4-dihydrodiol and with the parent hydrocarbon, the total number of tumours resulting from initiation by this diol is significantly greater ($P<0{\cdot}05$) than the number in the group treated with 7-methylbenz[a]anthracene. Other comparisons showed the 3,4-dihydrodiol to be significantly more active than the corresponding 1,2-, 5,6- and 8,9-dihydrodiols. In the control group of mice that were only treated with the phorbol ester, a single papilloma was observed at the 17th week, that later regressed. During the last 10 weeks of the experiment, 3 other papillomas developed in this group after the increased application of TPA.

The activities of benzo[a]pyrene in the suppression of sebaceous glands and the induction of hyperplasia in mouse skin are compared with those of 3 related dihydrodiols in Table II. Benzo[a]pyrene itself suppressed sebaceous glands and induced hyperplasia in a dose-dependent manner, and the hydrocarbon was clearly more active in both respects than any of the 3 related dihydrodiols. The 7,8-dihydrodiol showed some activity as a hyperplastic agent and in the suppression of sebaceous glands (Table II), but in both cases was considerably less active than benzo[a]pyrene. Hyperplasia was not induced by either the 4,5- or the 9,10-dihydrodiols, and neither decreased the numbers of sebaceous glands in areas of treated mouse skin.

Benzo[a]pyrene and the related *trans*-4,5-, 7,8- and 9,10-dihydrodiols were also

TABLE II.—*Development of epidermal hyperplasia and destruction of sebaceous glands in mouse skin after treatment with benzo[a]pyrene (BP) or with a related dihydrodiol*

| Compound | Dose* (μg) | No. of mice | Epidermal hyperplasia† | | Sebaceous glands‡ | |
|---|---|---|---|---|---|---|
| | | | Thickness§ | % of control | No. present | % destroyed |
| BP | 75·0 | 30 | 21·5±2·37 | 182·2 | 4·5±1·19 | 70·0 |
| | 112·5 | 20 | 29·4±4·18 | 249·2 | 1·3±0·93 | 93·3 |
| | 150·0 | 17 | 43·0±5·16 | 372·2 | 0·0 | 100·0 |
| *trans*-4,5-Dihydro-4,5-dihydroxy-BP | 75·0 | 30 | 11·0±0·78 | 93·2 | 14·6±2·10 | 2·7 |
| | 112·5 | 30 | 11·7±1·17 | 99·2 | 13·6±1·56 | 9·3 |
| | 150·0 | 15 | 12·6±2·27 | 106·8 | 13·7±2·48 | 9·3 |
| *trans*-7,8-Dihydro-7,8-dihydroxy-BP | 75·0 | 30 | 19·3±2·21 | 163·6 | 15·1±1·65 | — |
| | 112·5 | 30 | 19·7±2·29 | 167·0 | 14·9±3·62 | — |
| | 150·0 | 10 | 16·1±2·91 | 136·4 | 9·5±2·79 | 36·7 |
| *trans*-9,10-Dihydro-9,10-dihydroxy-BP | 75·0 | 30 | 11·2±1·22 | 94·9 | 14·8±1·47 | — |
| | 112·5 | 30 | 10·8±0·92 | 91·5 | 14·8±1·38 | — |
| | 150·0 | 19 | 10·9±1·05 | 92·4 | 14·6±1·48 | — |
| Acetone | 0·15 ml | 24 | 15·0±1·50 | 100·0 | 11·8±1·11 | — |

* Total dose applied in 3 applications, each of which contained the test compound as a solution in 0·05 ml acetone.
† In 12 microscopic fields as described in the text.
‡ In 12 microscopic fields of sections obtained as described in the text.
§ In arbitrary units.

tested for their abilities to induce sarcomas after a single s.c. injection (0·9 mg) into rats, and the results are given in Table III. Sarcomas were induced in 24/30 rats treated with the hydrocarbon itself, but the dihydrodiols were essentially inactive. No s.c. tumours were detected in the groups injected with either the 4,5- or the 9,10-dihydrodiols, and only one sarcoma was induced in the group of rats injected with the 7,8-dihydrodiol. All the tumours that occurred were first detected during the 6th month after the injection. They were classical s.c. tumours that evolved slowly, and the tumour-bearing animals that died or were killed 8–9 months after the start of the experiment carried large tumours. Postmortem and histological examinations showed that the tumours were fibrosarcomas with well-limited margins that had not metastasized; no other tumours were found in the rats when the experiment was terminated at 9 months.

TABLE III.—*Induction of sarcomas after s.c. injection of benzo[a]pyrene (BP) or a related dihydrodiol into Wistar rats**

| Compound | No. of animals | No. with sarcomas |
|---|---|---|
| BP | 30 | 24 |
| *trans*-4,5-Dihydro-4,5-dihydroxy-BP | 32 | 0 |
| *trans*-7,8-Dihydro-7,8-dihydroxy-BP | 32 | 1 |
| *trans*-9,10-Dihydro-9,10-dihydroxy-BP | 32 | 0 |

* Female rats received the test compounds as a single s.c. injection (0·9 mg) in 0·5 ml olive oil in the dorsal region.

### DISCUSSION

The results (Table I) when 7-methylbenz[a]anthracene and 4 related dihydrodiols were tested for tumour-initiating activity on mouse skin clearly show that the 3,4-dihydrodiol is more active in this respect than the parent hydrocarbon, which is itself more active than the related 1,2-, 5,6- and 8,9-dihydrodiols. The 10,11-dihydrodiol was not tested in these experiments because it was not available in sufficient quantity. The 1,2- and 8,9-dihydrodiols are, like the 3,4-dihydrodiol, non-K-region diols that possess an isolated double bond adjacent to the diol grouping and they can in theory be converted into vicinal diol-epoxides: a diol-epoxide of this type cannot be directly formed, however, from the K-region diol (the 5,6-dihydrodiol), which was essentially devoid of tumour-initiating activity in the present experiments (Table I). The high initiating activity of the 3,4-dihydrodiol is in agreement with other data from biophysical (Vigny *et al.*, 1977), biochemical (Tierney *et al.*, 1977) and biological (Malaveille *et al.*, 1977; Marquardt *et al.*, 1977) studies, which together strongly suggest that the metabolic activation of this hydrocarbon involves the 3,4-dihydrodiol and its conversion into the related vicinal diol-epoxides, the isomeric 3,4-dihydro-3,4-dihydroxy-7-methylbenz[a]anthracene, 1,2-oxides. 7-Methylbenz[a]anthracene is metabolized by mouse skin to a variety of hydroxylated products that include the 3,4-dihydrodiol (Tierney *et al.*, 1977) and, in the present experiments, the lower activity of the parent hydrocarbon than of the 3,4-dihydrodiol, may well reflect its metabolism by alternative pathways.

The results reported here for dihydrodiols derived from 7-methylbenz[a] anthracene and those obtained previously for diols derived from benzo[a]pyrene (Slaga *et al.*, 1976; Chouroulinkov *et al.*, 1976; Levin *et al.*, 1976) and from benz[a] anthracene (Levin *et al.*, 1978) show that comparative tests for mouse-skin tumour-initiating activity can assist in the identification of the dihydrodiols that are involved in the metabolic activation of the parent hydrocarbons.

In contrast, the results when the activities of benzo[a]pyrene and of its related dihydrodiols in the induction of hyperplasia and the suppression of sebaceous glands in mouse skin and in the induction of s.c. sarcomas in rats were examined were not so encouraging. In these short-term mouse skin tests (Table II), the 7,8-dihydrodiol of benzo[a]pyrene, which has shown biological activity equivalent to, or higher than, that of benzo[a]pyrene itself in a variety of other test systems (Malaveille *et al.*, 1975; Slaga *et al.*, 1976; Chouroulinkov *et al.*, 1976; Marquardt *et al.*, 1976), was clearly less active than the parent hydrocarbon. The activities of a range of polycyclic hydrocarbons in inducing hyperplasia and in suppressing sebaceous glands have been reported to correlate with their carcinogenic potencies (Bock & Mund, 1958; Guérin & Cuzin, 1961; Lazar & Chouroulinkov, 1974).

Somewhat similar results were obtained when the carcinogenic activities of benzo[a]pyrene and the related dihydrodiols were compared after their s.c. injection into rats (Table III). Although the 7,8-dihydrodiol produces tumours in mouse skin, and is as active in this respect as the parent hydrocarbon, it was very much less active than benzo[a]pyrene as an s.c. carcinogen in rats. Since it has been shown that short-term skin tests in mice are more relevant to the promoting activities of chemicals (Lazar & Chouroulinkov, 1974) and since the induction of s.c. sarcomas in rats appears to be related to the abilities of chemicals to act as complete carcinogens, the lack of activity shown by the 7,8-dihydrodiol of benzo[a]pyrene in these tests and the high activity that it has shown in other test systems suggests that it may be more effective as an initiating agent than as a complete carcinogen. However, this explanation requires experimental confirmation, and the differences between the activities of benzo[a]pyrene and of the related 7,8-dihydrodiol in the two test systems examined here may be due to pharmacokinetic differences in the metabolism of these compounds in these situations. Of the 3 test systems examined here, therefore, only the mouse skin initiation-promotion experiments have given results that are in broad agreement with the other available information that has identified the 7,8-dihydrodiol of benzo[a]pyrene and the 3,4-dihydrodiol of 7-methylbenz[a]anthracene as the dihydrodiols that are, through conversion into reactive bay-region vicinal diol-epoxides, important in the metabolic activation of these two carcinogenic hydrocarbons.

We wish to thank Paulette Ammari, Alan Hewer and Christine Walsh for their assistance with this work, which was supported in part by grants to the Chester Beatty Research Institute, Institute of Cancer Research: Royal Cancer Hospital, from the Medical Research Council and the Cancer Research Campaign.

### REFERENCES

BIGGER, C. A. H., TOMASZEWSKI, J. E. & DIPPLE, A. (1978) Differences between products of binding of 7,12-dimethylbenz[a]anthracene to DNA in mouse skin and in a rat liver microsomal system. *Biochem. Biophys. Res. Commun.*, **80**, 229.

BOCK, F. G. & MUND, R. (1958) A survey of compounds for activity in the suppression of mouse sebaceous glands. *Cancer Res.*, **18**, 887.

CHOUROULINKOV, I., GENTIL, A., GROVER, P. L. & SIMS, P. (1976) Tumour-initiating activities on mouse skin of dihydrodiols derived from benzo-[a]pyrene. *Br. J. Cancer*, **34**, 523.

CHOUROULINKOV, I., GENTIL, A., TIERNEY, B., GROVER, P. L. & SIMS, P. (1977) The metabolic activation of 7-methylbenz[a]anthracene in mouse skin: high tumour-initiating activity of the 3,4-dihydrodiol. *Cancer Lett.*, **3**, 247.

GROVER, P. L., HEWER, A., PAL, K. & SIMS, P. (1976) The involvement of a diol-epoxide in the metabolic activation of benzo[a]pyrene in human bronchial mucosa and in mouse skin. *Int. J. Cancer*, **18**, 1.

GUÉRIN, M. & CUZIN, J. (1961) Tests cutanés chez la souris pour déterminer l'activité carcinogène des goudrons de fumée de cigarettes. *Bull. Assoc. Franc. Cancer*, **48**, 112.

JEFFREY, A. M., JENNETTE, K. W., BLOBSTEIN, S. H. & 6 others (1976) Benzo[a]pyrene-nucleic acid derivative found *in vivo*: structure of a benzo[a]pyrene tetrahydrodiol epoxide-guanosine adduct. *J. Amer. Chem. Soc.*, **98**, 5714.

JEFFREY, A. M., WEINSTEIN, I. B., JENNETTE, K. W. & 5 others (1977) Structures of benzo[a]-pyrene-nucleic acid adducts formed in human and bovine bronchial explants. *Nature*, **268**, 348.

LAZAR, P. & CHOUROULINKOV, I. (1974) Validity of the sebaceous gland test and the hyperplasia test for the prediction of the carcinogenicity of cigarette smoke condensates and their fractions. In *Experimental Lung Cancer*. Ed. E. Karbe and J. P. Park, Heidelberg: Springer-Verlag. p. 383.

LAZAR, P., LIBERMANN, C., CHOUROULINKOV, I. & GUÉRIN, M. (1963) Tests sur le peau de souris pour la détermination des activités carcinogènes: mise au point methodologique. *Bull. Assoc. Franc. Cancer*, **50**, 567.

LEVIN, W., WOOD, A. W., RYAN, D., BUENING, M., JERINA, D. M. & CONNEY, A. H. (1978) Metabolic activation of 3-methylcholanthrene and its metabolites to bacterial mutagens. *Proc. Am. Ass. Cancer Res.*, **19**, 108.

LEVIN, W., WOOD, A. W., YAGI, H., JERINA, D. M. & CONNEY, A. H. (1976) (±) *trans*-7,8-Dihydroxy-7,8-dihydrobenzo[a]pyrene, a potent skin carcinogen when applied topically to mice. *Proc. Natl Acad. Sci. U.S.A.*, **73**, 3867.

MALAVEILLE, C., BARTSCH, H., GROVER, P. L. & SIMS, P. (1975) Mutagenicity of non-K-region diols and diol-epoxides of benz[a]anthracene and benzo[a]pyrene in *S. typhimurium* TA100. *Biochem. Biophys. Res. Commun.*, **66**, 693.

MALAVEILLE, C., BARTSCH, H., TIERNEY, B., GROVER, P. L. & SIMS, P. (1978) Microsome-mediated mutagenicities of the dihydrodiols of 7,12-dimethylbenz[a]anthracene: high mutagenic activity of the 3,4-dihydrodiol. *Biochem. Biophys. Res. Commun.*, **83**, 1468.

MALAVEILLE, C., TIERNEY, B., GROVER, P. L., SIMS, P. & BARTSCH, H. (1977) High microsome-mediated mutagenicity of the 3,4-dihydrodiol of 7-methylbenz]a[anthracene in *S. typhimurium* TA98. *Biochem. Biophys. Res. Commun.*, **75**, 427.

MARQUARDT, H., BAKER, S., TIERNEY, B., GROVER, P. L. & SIMS, P. (1977) The metabolic activation of 7-methylbenz[a]anthracene: the induction of malignant transformation and mutation in mammalian cells by non-K-region dihydrodiols. *Int. J. Cancer*, **19**, 828.

MARQUARDT, H., BAKER, S., TIERNEY, B., GROVER, P. L. & SIMS, P. (1978) Induction of malignant transformation and mutagenesis by dihydrodiols derived from 7,12-dimethylbenz[a]anthracene. *Biochem. Biophys. Res. Commun.*, **85**, 357.

MARQUARDT, H., GROVER, P. L. & SIMS, P. (1976) *In vitro* malignant transformation of mouse fibroblasts by non-K-region dihydrodiols derived from 7-methylbenz[a]anthracene, 7,12-dimethyl-benz[a]anthracene and benzo[a]pyrene. *Cancer Res.*, **36**, 2059.

MCCAUSTLAND, D. J., FISCHER, D. L., KOLWYCK, K. C. & 6 others (1976) Polycyclic aromatic hydrocarbon derivatives: synthesis and physico-chemical characterization. In *Polynuclear Aromatic Hydrocarbons: Chemistry, Metabolism and Carcinogenesis*. Eds. R. I. Freudenthal & P. W. Jones. New York: Raven Press. p. 349.

SIMS, P. (1967) The metabolism of 7- and 12-methylbenz[a]anthracene and their derivatives. *Biochem. J.*, **105**, 591.

SIMS, P. (1970) The metabolism of some aromatic hydrocarbons by mouse embryo cell cultures. *Biochem. Pharmacol.*, **19**, 285.

SIMS, P., GROVER, P. L., SWAISLAND, A., PAL, K. & HEWER, A. (1974) Metabolic activation of benzo[a]pyrene proceeds by a diol-epoxide. *Nature*, **252**, 326.

SLAGA, T. J., VIAJE, A., BERRY, D. L., BRACKEN, W., BUTY, S. G. & SCRIBNER, J. D. (1976) Skin tumor initiating ability of benzo[a]pyrene 4,5-, 7,8- and 7,8-diol-9,10-epoxides and 7,8-diol. *Cancer Lett.*, **2**, 115.

TIERNEY, B., ABERCROMBIE, D., WALSH, C., HEWER, A., GROVER, P. L. & SIMS, P. (1978) The preparation of dihydrodiols from 7-methylbenz[a]-anthracene. *Chem. Biol. Interact.*, **21**, 289.

TIERNEY, B., HEWER, A., WALSH, C., GROVER, P. L. & SIMS, P. (1977) The metabolic activation of 7-methylbenz[a]anthracene in mouse skin. *Chem. Biol. Interact.*, **18**, 179.

VIGNY, P., DUQUESNE, M., COULOMB, H. & 4 others (1977) Metabolic activation of polycyclic hydrocarbons. Fluorescence spectral evidence is consistent with metabolism at the 1,2- and 3,4-double bonds of 7-methylbenz[a]anthracene. *FEBS Lett.*, **75**, 9.

WOOD, A. W., LEVIN, W., LU, A. Y. H. & 6 others (1976) Mutagenicity of metabolically-activated benzo[a]anthracene 3,4-dihydrodiol: evidence for bay region activation of carcinogenic polycyclic hydrocarbons. *Biochem. Biophys. Res. Commun.*, **72**, 680.